# Galactoglucomannan Recovery with Hydrophilic and Hydrophobic Membranes: Process Performance and Cost Estimations

**DOI:** 10.3390/membranes9080099

**Published:** 2019-08-10

**Authors:** Basel Al-Rudainy, Mats Galbe, Frank Lipnizki, Ola Wallberg

**Affiliations:** Department of Chemical Engineering, Lund University, P.O. Box 124, SE-221 00 Lund, Sweden

**Keywords:** galactoglucomannan, lignin, lignin-carbohydrate-complex, lignosulfonates, ultrafiltration, fouling, cost-estimations

## Abstract

In this study, we compared the GR51PP (hydrophobic/polysulfone) membrane with a series of hydrophilic (regenerated cellulose) membranes with the aim of increasing the retention of products and decreasing membrane fouling. The raw material used was a sodium-based spent sulfite liquor from the sulfite pulping process of spruce and pine. The results show that the hydrophilic membranes were superior to the hydrophobic membranes in terms of higher fluxes (up to twice the magnitude), higher product retentions and less fouling (up to five times lower fouling). The fouling was probably caused by pore blocking as observed in earlier studies. However, the hydrophilic membranes had a lower affinity for lignin, which was indicated by the lower retention and fouling. This also resulted in a separation degree, which was higher compared with the hydrophobic membrane, thus yielding a higher galactoglucomannan (GGM) purity. 2D HSQC NMR results show that no major structural differences were present in the hydrophilic and hydrophobic retentates. A techno-economical evaluation resulted in the RC70PP being chosen as the most cost-efficient membrane in terms of flux and product recovery.

## 1. Introduction

Spent sulfite liquor (SSL) is a waste stream that is generated in the sulfite pulping process, where it is usually incinerated for the production of power and heat [1]. The solids content of SSL is typically 8–14 wt % and the main components are sugars (mono- and polysaccharides), lignosulfonates, extractives, and pulping chemicals [1,2]. Studies concerning the different wood components have also shown that a part of the lignin is covalently bound to polysaccharides, forming the so-called lignin-carbohydrate-complexes (LCC) [3]. This makes the different plant-based waste streams very complex in terms of composition, which depends both on the used biomass raw material and the extraction method. 

SSL from spruce contains mainly lignosulfonates (LS), galactoglucomannan (GGM), arabinoglucuronoxylan and LCC [4]. It was further discovered by Giummarella et al. [5] that the main bonds between lignin and a carbohydrates are benzyl ether to xylan and phenyl-glycosidic to mannan. Other possible bonds, which have been established, exist between arabinan or galactan and lignin [4,6]. Du et al. [7] made similar observations, and found that the major LCC formations are found to be between lignin and mannan, xylan or glucan. The LCCs were shown to have a high molecular weight and were insoluble in dioxane/water mixtures. 

Potential applications for GGM, lignin, and LCCs have been highlighted in many studies. The mentioned components could be used for the production of gas-barrier films, polymeric surfactants and as drug carriers [8,9]. This makes the isolation and fractionation of these compounds very important for the future development of innovative products.

Membrane filtration is a technology that has shown promise in this field. Many different studies have been conducted on wood-based process waters to fractionate and isolate these components. Al Manasrah et al. [10] recovered 70% of the GGM in spruce saw dust hot-water extracts with a GGM purity of 63% at a volume reduction (VR) of 86% using ultrafiltration membranes. Duval et al. [11] used regenerated cellulose membranes with a molecular weight cutoff between 1 and 30 kDa to fractionate lignosulfonates with high polydispersity. The authors’ results also showed that it was possible to obtain retentates with low polydispersity index and high yields. Bhattacharya et al. [12] used ultrafiltration membranes to recover lignosulfonates from SSL. The authors compared different membrane materials, such as polysulfone, regenerated cellulose, and fluoropolymer. The results showed that a high molecular weight cutoff (MWCO) polysulphone membrane was optimal in terms of flux and rejection. In addition, a tendency for fouling of the polysulphone membranes as part of their flux vs time figures was also noticed; however, the differences between the membranes in terms of fouling were not discussed. 

LCCs have been shown to be difficult to isolate using membrane filtration [13]; the reason is the narrow MW difference between the GGM and the LCCs and, in some cases, the formation of a fouling layer on the membrane surface that increases the retention of all solutes and thus prevents fractionation. Membrane filtration has been shown to be successful in concentrating and purifying the SSL from pulping chemicals and other low MW compounds; however, for the separation of the components other methods have to be used.

To solve the fractionation problem, anti-solvent precipitation has been shown to be an effective method. Compared to membrane filtration, anti-solvent precipitation utilizes the difference in solubility of different solutes and thus can separate components with narrow molecular weight differences [4,14,15]. This has been used by Song et al. [14] to separate GGM from a spruce hot-water extract with ethanol as anti-solvent. The efficiency of the precipitation and yield of product was high; however, the amount of ethanol consumed was around 90% of the in-going raw material feed; thus, the conclusion was that the separation process is too costly. Zasadowski et al. [15] separated GGM from lignin using the same process and obtained results showing that acetone was a more efficient anti-solvent. Acetone has a lower dielectric constant (lower polarity), which lowers the polarity of the bulk solvent at a reduced addition compared to ethanol [4]. The process could be further improved by concentrating the raw material before the anti-solvent addition, causing further reduction in solvent requirement and increasing the product yield. In this case, the combination of membrane filtration and anti-solvent precipitation is beneficial. Membrane filtration is used to concentrate the SSL solution and to remove pulping chemicals through diafiltration. The concentrated and purified SSL is then fed to the anti-solvent process to separate the different solutes. This process requires the membrane filtration to be efficient in terms of a high flux, high retention of products, and low fouling.

In a previous study, the isolation of GGM, lignin, and LCCs from spent sulfite liquor, and the minimization of membrane fouling was examined [13]. Three hydrophobic membranes with molecular weight cutoffs (MWCOs) of 100, 50, and 25 kDa, respectively, were screened for this purpose. Flux and retention for the 100 and 50 kDa membranes were identical, which indicated that the separation properties of the membranes were determined by the fouling layer on the membranes and not the pore size. The membrane with the 50 kDa MWCO performed best in terms of flux and retention of product and was less prone to fouling. Prefiltration of the raw material using microfiltration increased the capacity of the 50 kDa MWCO membrane and the effect of membrane fouling was nearly eliminated. The retention of the product was, however, high during the microfiltration. This resulted in high losses of product during the prefiltration stage. Dead-end filtration has been shown to be a promising alternative to microfiltration, because of its potentially lower loss of product. However, dead-end filtration requires the use of filter-aids (e.g., diatomaceous earth) which could affect the pulp mill recovery process in the case of any membrane filtration failures. 

Another way of decreasing membrane fouling is by increasing the hydrophilicity of the membrane material. Lignin as a hydrophobic macromolecule usually adsorbs to hydrophobic membrane materials through non-covalent interactions [16]. This adsorption can thus occur on the surface of the membrane, inside the membrane pores and cause blocking and non-reversible fouling [13,17]. Li et al. [17] showed that membrane fouling occurred during ultrafiltration of kraft-lignin using polysulfone membranes. The observed effects were a declining flux over time and the formation of a yellow coating layer on the membrane surface that could not be removed either by water or by a 0.1 M NaOH solution. As a reference, regenerated cellulose membranes were used in the same study. The result was that the permeability was unaffected by exposing the membrane to a kraft-lignin solution and no yellow coating was formed on the membrane surface. This showed that by switching to a hydrophilic membrane, fouling could be decreased or avoided. A similar study was carried out by Puro et al. [18] where a method was developed and evaluated to identify foulants during ultrafiltration of ground wood mill circulation water. In the study, three membranes were used: two hydrophobic (polyethersulfone and polyamide) and one hydrophilic (regenerated cellulose). The major foulants detected were fatty acids, resins, and lignans, all of which are mainly hydrophobic compounds. Consequently, the hydrophobic membranes were heavily fouled while the hydrophilic membrane was mildly fouled. This was also confirmed from a seven day long flux measurement where the hydrophobic membranes experienced a decrease from 200 to 30 L/m^2^ h, while the hydrophilic membrane maintained a constant flux of around 360 L/m^2^ h.

The aim of this study was to compare the optimal hydrophobic membrane found in a previous study [13] to regenerate cellulose membranes with different MWCOs. The difference between the retentates obtained using the hydrophobic and hydrophilic membranes was examined and a cost estimation was used to determine which membrane was the most cost efficient.

## 2. Materials and Methods 

### 2.1. Raw Material

The raw material used was a sodium-based spent sulfite liquor, which was extracted from the first step in a two-step softwood (60% *Picea abies* and 40% *Pinus sylvestris*) pulping process (Domsjö Fabriker, Örnsköldsvik, Sweden).

### 2.2. Equipment and Experimental Procedure

#### 2.2.1. Membranes and Membrane Filtration Set-Up

The hydrophilic membranes used in this study are listed in Table 1. The hydrophobic membrane used in our previous study is also included as reference. All of the membranes withstand 50 °C solutions with pH between 2–10 and operating pressures of 1 to 10 bars. 

**Figure 1:**
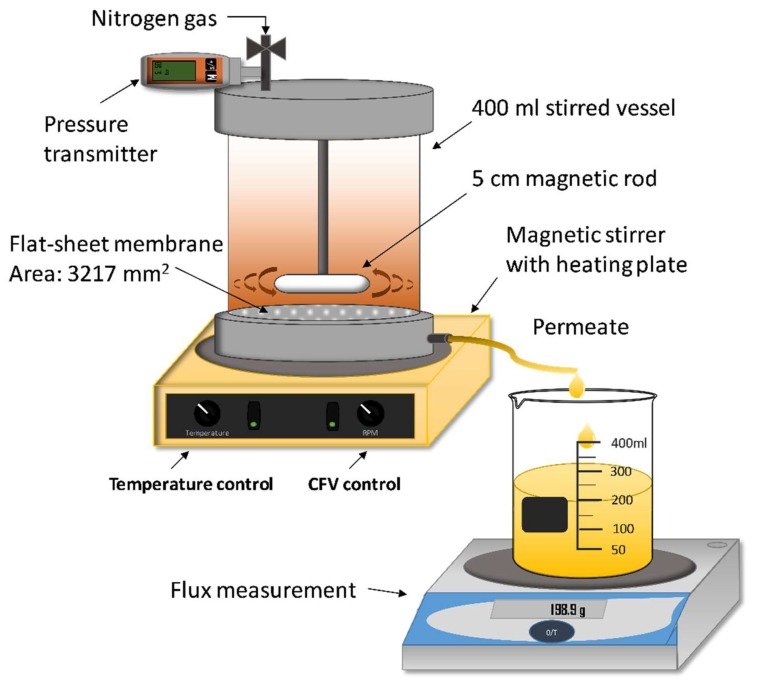
Illustration of the setup used for the membrane filtration. CFV = cross-flow velocity.

The studies were done using a 400 mL stirred vessel shown in Figure 1. The vessel was heated using a heating plate (MR2002, Heidolph Instruments GmbH & Co.KG, Schwabach, Germany) and the cross-flow velocity (CFV) was controlled by changing the speed of the internal magnetic stirrer. The conversion between CFV and speed of the internal magnetic stirrer was implemented according to previous work [13]. The pressure was monitored using a digital pressure gauge (DCS40.0AR, Trafag AG, Bubikon, Switzerland) and changed through a valve connected to a nitrogen gas line. The flux was measured using a balance (PL6001-l, Mettler Toledo Inc., Columbus, OH, USA). 

#### 2.2.2. Influence of Volume-Reduction on Flux and Retention (Concentration Study)

The concentration studies were performed according to parameters derived in previous work [13]. The temperature, CFV, transmembrane pressure (TMP), and volume reduction were, 50 °C, 0.5 m/s, 5.5 bar and 90%, respectively. The process was started by cleaning a new membrane according to Section 2.2.3 and the pure water flux (PWF) was measured thereafter. The SSL was then filtered through 250, 180, and 45 µm filter trays (U.S.A. Standard Testing Sieve, WS Tyler Inc., Mentor, OH, USA) in series and loaded into the 400 mL vessel (Figure 1). The solution was then heated with the heating plate and pressure was applied when a temperature of 50 °C was reached and the flux recorded. When the specified volume reduction (ratio between the permeate and total initial volume) was achieved, the process was stopped, and samples of both permeate and retentate were taken. The membrane was cleaned again according to Section 2.2.3 and the PWF measured once more. 

#### 2.2.3. Membrane Cleaning and Fouling Calculations

The concentration studies began and ended with a cleaning using a 0.04 wt % acid detergent solution (Ultrasil 73, Ecolab AB, Älvsjö, Sweden) followed by measuring the PWF. A detergent volume of 350 mL was loaded into the module and heated to 50 °C under constant stirring (CFV of 0.5 m/s) and was left for around 1 h. The solution was then filtered through the membrane until a volume reduction of 50% was achieved (175 mL of the solution was passed to the permeate). The remaining retentate was discarded and the membrane was washed using 2 module volumes of deionized water at room temperature. The module was then filled with deionized water and the flux was measured at 20 °C, 0.5 m/s in CFV, and a trans-membrane pressure of 5.5 bar. The fouling degree was calculated from the flux of the deionized water (also called PWF) before and after the experiments, as per expression:

(1) $$FoulingDegree(\%)=100*\frac{PWF_{\mathrm{Before}}-PWF_{\mathrm{After}}}{PWF_{\mathrm{Before}}}$$

### 2.3. Analysis

#### 2.3.1. Lignin Content

The concentration of lignin in solution was determined using a spectrophotometer (Shimadzu UV-1800, Kyoto, Japan). The wavelength was set to 234 nm and the extinction coefficient used was 31.6 L/(g cm) [13].

#### 2.3.2. Ash and Total Dry Content

The total dry content was determined by weighing 3 mL of sample in a ceramic crucible and drying it in an oven (Heraeus, Heraeus Holding GmbH, Hanau, Germany) at 105 °C for 24 h after which the sample was weighed again. After determining the total dry content, the samples were placed in a furnace (B150, Nabertherm GmbH, Lilienthal, Germany) where they were ashed at 575 °C for 4 h. The ash content was determined by weighing the samples after they had cooled down to room temperature in a desiccator. 

#### 2.3.3. Hemicellulose and Acid-Insoluble Solids

The hemicellulose content was determined according to a standardized National Renewable Energy Laboratory (NREL) method [19]. The samples were acid hydrolyzed by adding 750 µl 72% sulfuric acid to 10 mL sample and autoclaved (Systec DX 150, Wettenberg, Germany) at 121 °C for 1 h. The samples were thereafter filtered to remove the acid-insoluble solids. The filter was then dried and weighed to determine the acid insoluble content. The filtrate was diluted with deionized water and analyzed with high-performance anion-exchange chromatography (HPAEC). The HPAEC system consisted of an ICS-5000+ DC (Dionex, Sunnyvale, CA, USA) equipped with pulsed amperometric detection running at a compartment temperature of 30 °C. The different monosaccharides were separated using a Carbo Pac PA1 analytical column where the eluent was deionized water at a flow of 1 and 0.5 mL/min 200 mM sodium hydroxide solution post-column addition. The injection volume was 10 µl and the standards used were L-arabinose, D-galactose, D-glucose, D-xylose, and D-mannose all manufactured by Fluka Chemie AG (Buchs, Switzerland). The amount of hemicellulose was determined after anhydro corrections of 0.90 for hexoses and 0.88 for pentoses. 

#### 2.3.4. Size-Exclusion Chromatography 

The molecular weight distribution was determined using size-exclusion chromatography (SEC). The system consisted of a controller (Waters 600E, Waters, Milford, MA, USA) connected to a refractive index detector (Waters 2414 Differential Refractometer) and UV detector (Waters 486 Tunable Absorbance Detector) set to 234 nm. The column used was a TSKgel (G4000PWXL, TOSOH Bioscience GmbH, Griescheim, Germany) and the eluent was deionized water, which was pumped using a Waters 600 gradient pump at a flow rate of 0.5 mL/min and degassed using a Waters in-Line degasser. The injection volume was 20 µL, which was performed using an autosampler (Waters 717 plus autosampler). The standards used were polyethylene glycol (400 Da, Merck Schuchardt OHG, Germany) and dextran (2000, 500, 100, 150, 60, 10 and 4 kDa Merck Schuchardt OHG, Germany). The same standards, instrument parameters and column were used for an alkali SEC (100 mM NaOH eluent) with a Shimadzu (Shimadzu Corp., Kyoto, Japan) system (SIL-10AXL autosampler, LC-10AT pump, CTO-10A column oven, RID-10A refractive index detector and SPD-10AV UV-detector).

#### 2.3.5. Two-Dimensional Nuclear Magnetic Resonance Spectroscopy (2D-NMR)

For the feed sample 1 mL was dried at 50 °C for 48 h and dissolved in 0.6 mL of deuterium oxide (Sigma-Aldrich Co., St. Louis, MO, USA) or 0.6 mL of a mixture of D_2_O and D_6_-DMSO (ratio of 3:5). The same sample preparation was used for the retentates; however, the amount of sample dried was 200 µL instead of 1 mL. The instrument used for the heteronuclear single quantum coherence spectroscopy 2D-NMR was a Bruker Avance III HD 500 MHz spectrometer (Bruker BioSpin GmbH, Karlsruhe, Germany). A 5 mm broadband (BBO) probe was used together with a Z-gradient coil. The data was acquired with the pulse program “hsqcetgpsisp.2” with the following settings: 136 scans, 1.5 s relaxation delay, 10.3 µs pulse length, 11 ppm spectral width, 1538 FID size and frequency discrimination in F1 e/a. The data was illustrated, processed, and evaluated using MestReNova 12 (Mestrelab Research S.L., Santiago de Compostela, Spain). Processing methods used were base-line correction and phase correction. Semi-quantative calculations and assignments were done according to methods used in the literature [4,20,21,22,23].

## 3. Results and Discussion

### 3.1. SSL Composition

**Figure 2:**
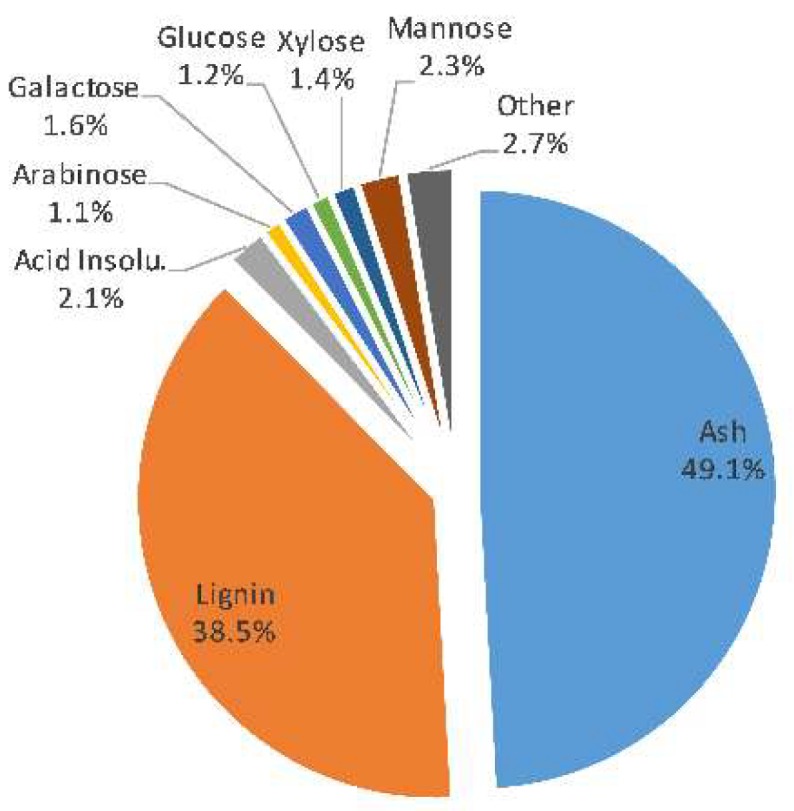
Composition of the untreated spent-sulfite-liquor raw material.

The results for the raw material composition used in this study are presented in Figure 2. According to these results, approximately 49.1% of the total dry content of the solution (TDS) of 80.1 g/L is composed of pulping chemicals. It is known that sodium-based SSL solutions contain sodium lignosulfonates [8,24]; thus, some of the ash content was a result of the decomposition of the lignosulfonates. Lignin had the second highest proportion of around 38.5% followed by the mono- and polysaccharides of which the total was 7.6%. GGM was the hemicellulose having the highest proportion of around 67% of the total hemicelluloses followed by xylan, and lastly arabinan.

**Figure 3:**
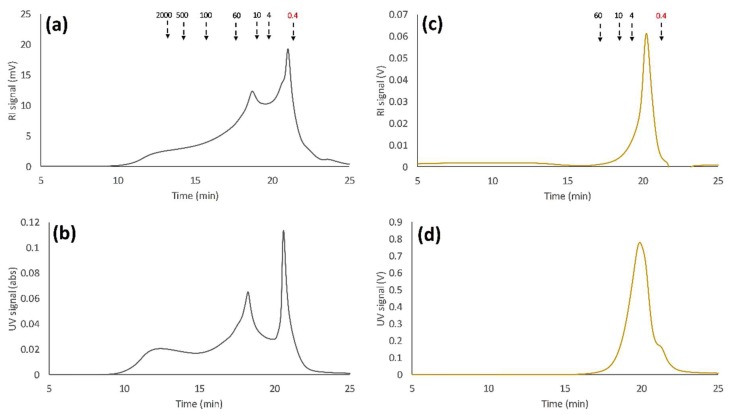
Size-exclusion chromatography (SEC) of the untreated spent-sulfite-liquor raw material; where (**a**,**b**) are the RI and UV detector response respectively using water as eluent. (**c**,**d**) are the RI and UV detector response respectively using 100 mM NaOH as eluent. Arrows point to the peak maximum of the various dextran standards, except for the lowest MW standard (0.4 kDa) which was PEG.

The molecular weight distribution for the untreated SSL solution was determined using SEC as shown in Figure 3. The results show that the major peaks are in the range between 500 and 0.4 kDa (Figure 3A,B), which is in agreement with previous observations using water as an eluent [4]. Although the signal was the strongest in the lower range, the high polydispersity reached molecular weights of up to 2000 kDa. This has not been seen previously, and it appeared only using a high-resolution SEC column. The refractive index (RI) and ultraviolet (UV) signals coincided in the entire measured MW region. This is an indication of the existence of lignin-carbohydrate-complexes [25]. However, previous work has shown that lignin-free polysaccharides exist in the MW region of 20 to 4 kDa. This was not visible in the untreated sample, presumably because of the overlapping signals with lignin or lignin-carbohydrate-complexes. 

Previous reports have shown that lignosulfonates were ion excluded when water was used as an eluent during SEC measurements [26]. This resulted in the early elution of the solutes and high MW determinations. The ion-exclusion effect was suppressed by using an electrolyte solution as the eluent. The sample was therefore separated with a 100 mM sodium hydroxide solution as SEC eluent (Figure 3C,D), which has been reported to be a reliable electrolyte for the MW determination of both lignin and lignosulfonates [27]. The results in Figure 3C,D show that the MW of the solutes was actually lower than that observed using water as an eluent (Figure 3A,B). This indicates that ion-exclusion effects were suppressed; in addition, it also explains the low lignin retention observed in our previous study [13]. 

### 3.2. Influence of Volume Reduction on Flux and Retention

**Figure 4:**
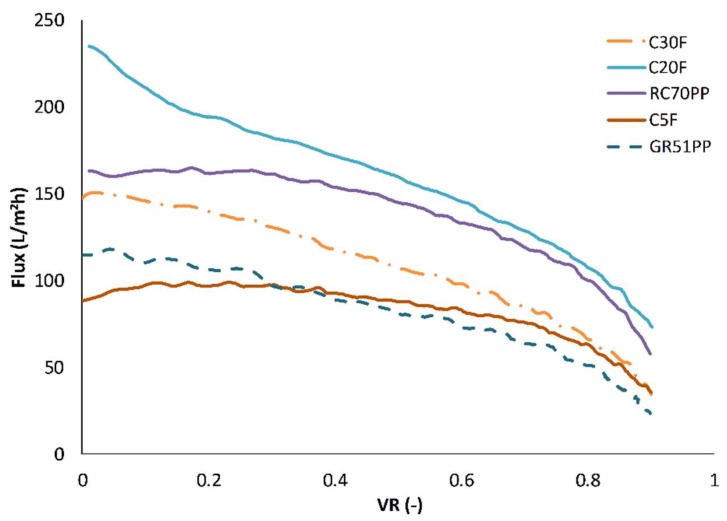
Flux vs volume reduction (VR) curves for the different concentration studies performed using the hydrophobic and hydophilic membranes. The temperature, trans-membrane pressure and cross-flow velocity were 50 °C, 5.5 bar, and 0.5 m/s, respectively.

**Figure 5:**
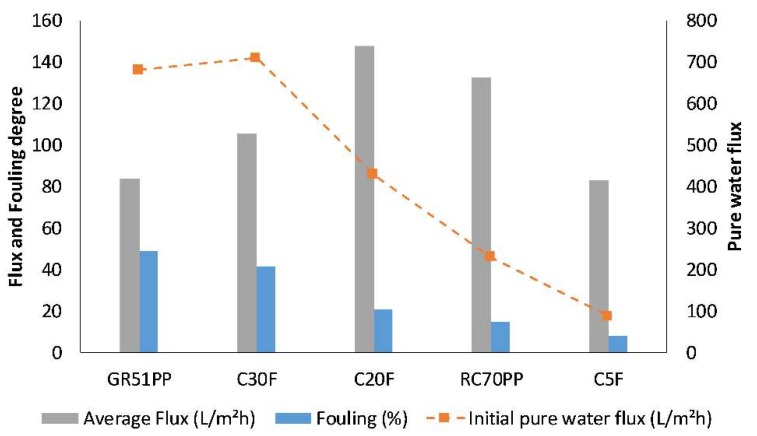
The average flux, fouling degree, and initial pure water flux (new and cleaned membrane) for the studied membranes.

In order to compare the hydrophilic membranes with the optimal hydrophobic membrane from our previous study [13], concentration studies were performed with the hydrophilic membranes using previously chosen parameters of 0.5 m/s CFV, 5.5 bar TMP, and 50 °C and a volume reduction of 90%. The flux vs volume reduction (ratio between the volume of permeate and feed) for the different membrane studies is shown in Figure 4. The hydrophobic membrane (GR51PP) had an almost linear decline of flux with increasing volume reduction. The same trend was observed for the more open hydrophilic membrane (C30F), while for the dense membranes (RC70PP and C5F) the flux was in pseudo steady state up to a volume reduction of 50%–60% thereafter it strongly decreased. The C20F membrane had a sharp decrease in the beginning that transitioned into a pseudo steady state mode and ended with a profile that followed the RC70PP and C5F. The sharp decrease in the beginning was probably caused by the formation of a cake on the surface as seen and explained by Brião et al. [28] and Hwang et al. [29]. The C30F had a larger MWCO than the C20F: thus, it was expected to have an overall higher flux based on this and the higher PWF seen in Figure 5. This did not occur, which implied that pore plugging was the probable main reason for this instantaneous decrease of flux and capacity [29]. This was also indicated by the fouling degree (Figure 5), which was higher for the C30F compared with that of the C20F. It is known that the raw material contains compounds with molecular weights ranging from approximately 60 to 0.4 kDa (Figure 3C). It has also been shown in our previous work [13] that a hydrophobic 25 kDa MWCO membrane became pore blocked causing a flux of virtually zero using the same raw material. Considering that the C30F membrane was in the same range of MWCO, this can support the concept of pore blockage as the main fouling mechanism. However, the flux was not zero, which probably was because of the slightly higher MWCO, but also because of the hydrophilic nature of the membrane, which can lead to low lignin adsorption [16]. 

**Figure 6:**
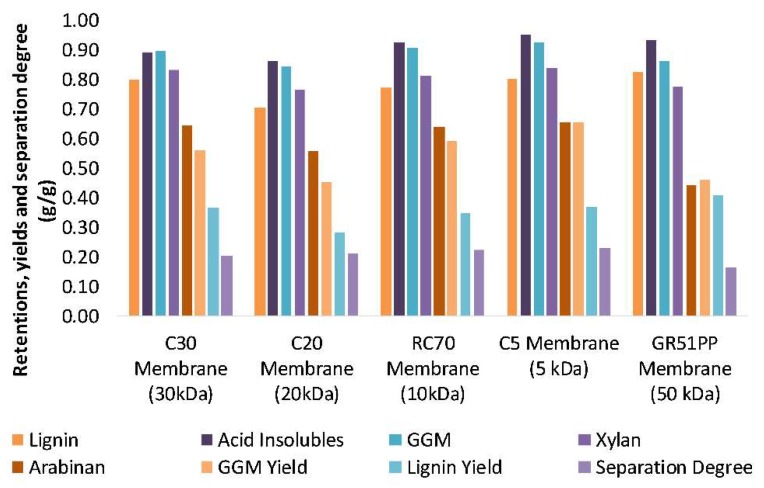
The average membrane retention, yield, and separation degree of the solutes from a volume reduction of 0% to 90%.

These results were also reflected in the average retention of the different compounds during the concentration study as seen in Figure 6. The average retention for lignin was similar for both the hydrophobic 50 kDa membrane and the hydrophilic 5 kDa membrane even though the MWCO differed by an order of magnitude. The retention for the polysaccharides behaved as expected, while the retention was higher for the denser membranes independently of the hydrophilicity of the membrane. Thus, there was no clear indication of any interaction between the membranes and the polysaccharides. The interaction between lignin and the hydrophobic membrane and the low retention of polysaccharides (high MWCO) clearly resulted in a separation degree (as defined by the concentration of GGM divided by the sum of the lignin and the GGM concentrations [4]), which was lower compared to the hydrophilic membranes (Figure 6). These observations clearly showed that the hydrophilic membranes in this case are superior when it comes to performance, separation, low fouling, and product yields. 

**Figure 7:**
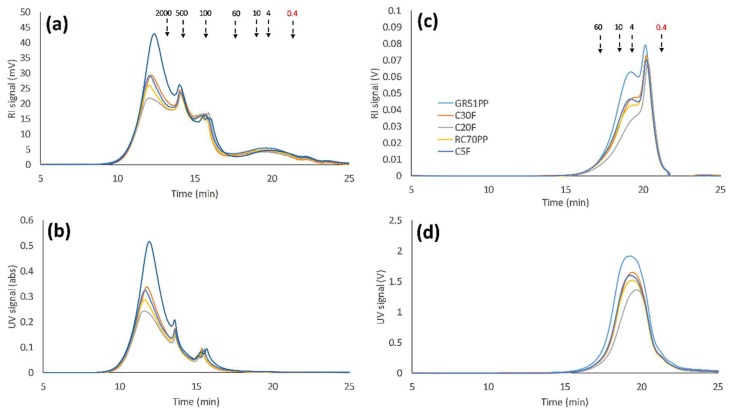
Size-exclusion chromatography of the volume-reduced retentates (90%) for all of the studied membranes; where (**a**,**b**) are the RI and UV detector response respectively using water as eluent. (**c**,**d**) are the RI and UV detector response respectively using 100 mM NaOH as eluent. Arrows point to the peak maximum of the various dextran standards, except for the lowest MW standard (0.4 kDa) which was PEG.

The molecular weight distributions for the membrane filtration retentates were determined using SEC and are presented in Figure 7. The peaks showing the lignin-free hemicelluloses were now visible between the standards 10 and 4 kDa using the RI detector (Figure 7A). These peaks did not have a corresponding signal using the UV detector (Figure 7B), which was expected since the polysaccharides were missing the lignin fragments that give rise to these signals [4,30]. The intensity of the peaks appeared to follow the retention of the components as seen in Figure 6. The highest lignin retention was around 0.82 for the 50 kDa hydrophobic membrane, which was in agreement with the highest intensity seen in the UV and RI curves in Figure 7A,B. The lignin retention was slightly lower (0.80) and equal for the 30 and 5 kDa hydrophilic membranes; thus, the SEC curves have approximately the same intensities. The 5kDa hydrophilic membrane had the highest GGM retention, which was complemented with a higher intensity for the peaks that correspond to the lignin-free hemicellulose. The major change observed compared with SEC of the untreated SSL (Figure 3A,B) was the shift of the majority of the peaks from a lower range of MW to a higher range. In Figure 3, lignin signals can be seen in the lower MW range, which do not appear in the results in Figure 7. The lignin retention was greater than zero, and thus, lower MW lignin would be likely to appear in Figure 7 regardless of which membrane was used for filtration. The MW of the lignin was also higher according to Figure 7. This would usually cause lignin to have a higher retention compared with the other components. This, however, did not occur and the retention was lower as seen in Figure 3. As discussed in Section 3.1, this is likely to be caused by ion exclusion effects, which may result in higher MW. The same samples were analyzed using alkali SEC, presented in Figure 7C,D. These results show that the most open membranes (GR51PP and C30F) had the highest intensity of most components, which was probably caused by a higher degree of fouling (Figure 5). This was especially observable for the components with a MW lower than 4 kDa, which had a higher retention for the membranes with a MWCO higher or equal to 30 kDa. 

### 3.3. Differences between the GR51PP and RC70PP Retentates

**Figure 8:**
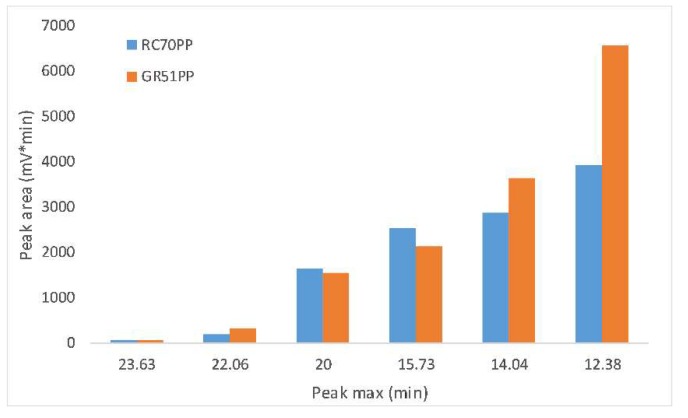
Comparison of the size-exclusion chromatography peak areas (RI) for the results shown in Figure 7 (RC70PP and GR51PP membranes).

In order to compare the difference between the hydrophilic and hydrophobic membrane, the retentates from both membrane types were compared. The hydrophilic membrane chosen was the one found optimal in Section 3.4 (RC70PP) and the hydrophobic was the GR51PP. From previous results, it was concluded that the hydrophilic membrane had a higher retention for the polysaccharides compared with the hydrophobic membrane and that the lignin retention was the opposite. This was also indicated by the SEC results shown in Figure 7 and illustrated in Figure 8. The peaks were gathered in six major parts. Three peaks were in the region 12 to 16 min, which also had a UV response (related to lignosulfonates and LCCs). The second three major peaks were in the region 16 to 24 min, which were lignin-free monosaccharides and polysaccharides given the lack of any UV response [30]. The results in Figure 8 show that the lignin-free region was the highest for the hydrophilic membrane (RC70PP). This is caused by the retention for the polysaccharides being higher for this membrane. However, the peak with a maximum intensity around 15 min also yielded a higher intensity using the hydrophilic membrane compared with the hydrophobic since the polysaccharide content is higher in this retentate and the peak has a UV response. This indicates that the hydrophilic retentate contained a higher amount of LCCs. LCCs could also be a part of the peaks in the high MW region (<15 min); however, because of the high amount of lignin in these peaks, this could not be confirmed. 

**Figure 9:**
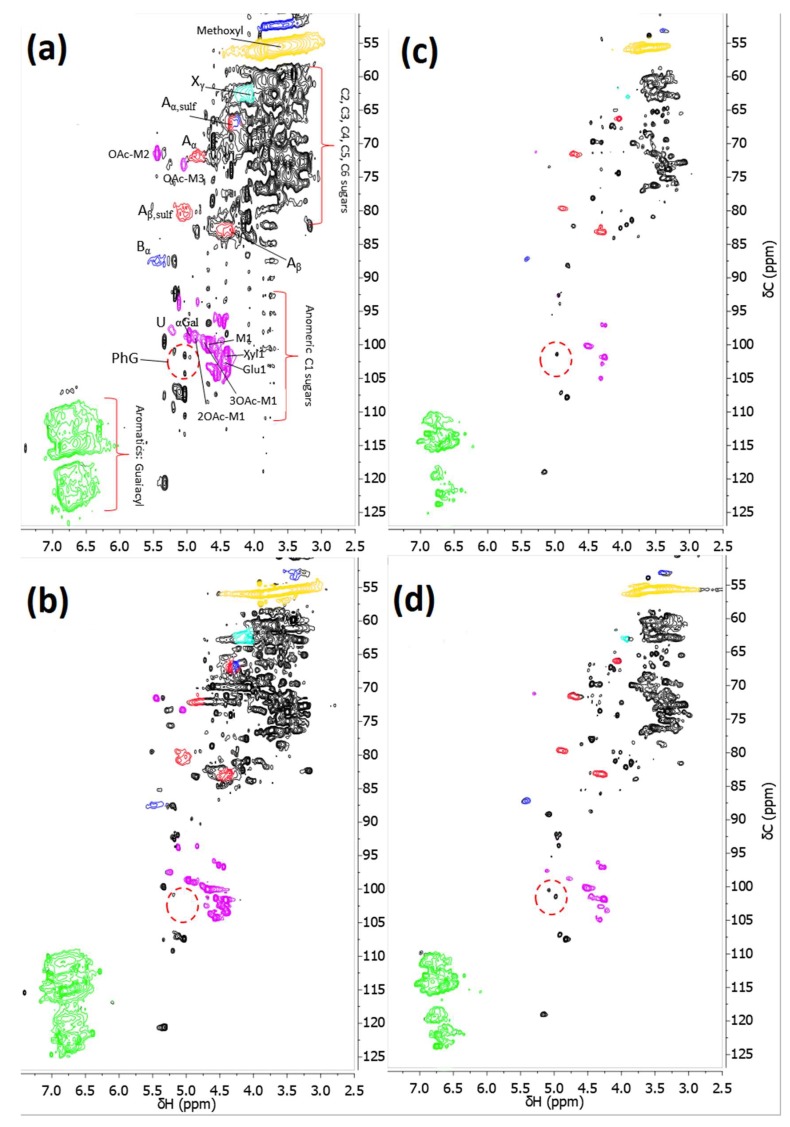
2D HSQC NMR using D2O (spectrum **a** and **b**) and a mixture of D2O and d6-DMSO (spectrum **c** and **d**) as solvent. Spectrums **a** and **c** represent the RC70PP retentate while spectrums **b** and **d** represent the GR51PP retentate. The identified lignin bonds were: A_x,y_ (B-aryl-ether bonds, red), B_x,y_ (phenylcoumaran, blue), X_γ_ (cinnamyl alcohol, light blue), PhG (phenyl glycosidic LCC bonds, red circle) and the aromatics in the guaiacyl units (green). The identified polysaccharides and uronic acids (purple) were: MX (mannopyranose), XOAc-MX (O-acetylated mannopyranose), XylX (xylopyranose), GluX (glucopyranose), α-Gal (galactopyranose), and U (glucuronic acid). The notations x and y are the x-carbon and sulfonation respectively.

It has previously been shown with 2D HSQC NMR that the major bonds between the polysaccharides and lignin were phenyl glycosyl (PhG) and benzyl ether bonds (BE1 (both hexoses and pentoses) and BE2 (mostly to xylan) [4]. Signals for benzyl ester bonds were also visible; however, because of overlapping signals these could not be confirmed. Our previous study also indicated the existence of sulfonated lignin in the raw material [8]. Thus, the signals for the benzyl ether bonds would overlap as well. The retentates in this study were analyzed employing 2D HSQC NMR using D2O and d6-DMSO as solvents and references (Figure 9). The results showed that a shift of the peaks occurs on the proton axis when changing from D2O to D_6_-DMSO. However, the relations between the peaks remain the same for the major interesting peaks. The intensity also becomes lower using D_6_-DMSO, which may be caused by the lower solubility of the polysaccharides in D_6_-DMSO (that has a lower dielectric constant than water) [4]. As a result, acetylated mannopyranose (2OAc-M1 and 3OAc-M1), alpha-galactopyranose (α-Gal), and uronic acids (U) were not visible. Possible phenyl glycosidic LCC linkages (PhG) were visible (highlighted with a red circle in the anomeric C1 sugars region) in all of the spectrums. The ratios between the sum of PhG volume integrals and mannopyranose (M1) volume integral (Figure 9c,d) for the RC70PP and GR51PP retentates were 0.20 and 0.15, respectively, indicating that a higher amount of LCCs was in the retentate after filtering, utilizing the hydrophilic membrane. Regarding the GGM composition, the α-galactopyranose groups content linked to the glucomannan backbone was around 16% of the total non-acetylated mannan for both retentates. This corresponds to a Gal:Man ratio of 0.4:3.0 which is in agreement with our previous study [4]. No other major differences in acetylated GGM were observed given that the total acetylated mannan was approximately 14% of the total mannan for both retentates. Semi-quantification of the visible lignin peaks (β-aryl-ether (A), β-5 (B), and methoxyl) was difficult brought about by the highly overlapping regions. However, by comparison between the two retentates the following values were obtained (per 100 aromatic rings): for the total β-O-4 (sulfonated and non-sulfonated) we obtained 37 and 40 for the RC70PP and GR51PP, respectively. Regarding phenylcoumaran (β-5) the values were 10 and 8, and for methoxyl the values were 100 and 86. The values are in the same range as seen in the literature for spruce [5] and the difference between the retentates was negligible.

### 3.4. Optimal Membrane and Cost Estimates

In order to decide which membrane is the most optimal, several considerations are required. The decision should rely on a membrane that would deliver the smallest full-scale plant but also has the highest GGM production and separation degree. In terms of separation degree, the differences between the hydrophilic membranes were very small (Figure 6); thus, this does not have to be accounted for. The flux and GGM production were different and thus a method was needed to choose the optimal membrane. This was done by performing plant cost estimations on the different membranes. The assumptions made in these calculations (Table 2) were derived from others [31], based on experience and contact with manufacturers. The cost estimates were accomplished assuming that the plant would be based on a spiral module with 48 mil spacer (Alfa Laval Nordic A/S, Søborg, Denmark). The maximum operating conditions for the module, such as maximum pressure drop, were derived from Alfa Laval’s datasheet and are presented in Table 2. Surrounding infrastructure was assumed to be a part of the existing plant where the unit was to be installed. In a typical plant for concentration duties, two kinds of pump are used. A feed pump is used to feed the untreated solution and increase the inlet pressure to the required operating pressure. A recirculation pump is also used to compensate for pressure drops and maintain a constant operating CFV. The electricity required per cubic meter of permeate to run these pumps was calculated according to Equation (2) (feed pump) and Equation (3) (recirculation pump) [32]:

(2) $$W_{\mathrm{feed}}=\frac{P_{\mathrm{inlet}}}{\eta VR}\;\left[\mathrm{kWh}/m^{3}\mathrm{permeate}\right]$$

(3) $$W_{\mathrm{recirculation}}=\frac{\Delta P_{f}Q_{\mathrm{housing}}}{\eta \left(J_{\mathrm{avg}}A_{m}\right)}\;\left[\mathrm{kWh}/m^{3}\mathrm{permeate}\right]$$

where *W*_feed_ and *W*_recirculation_ are the required electrical energy, *P*_inlet_ is the inlet pressure, *η* is the pump efficiency, VR is the volume reduction, Δ*P*_f_ is the pressure drop, *Q*_housing_ is the feed flow to one module, *J*_avg_ is the average flux, and *A*_m_ is the membrane area in one module. 

The results from the cost estimations (Table 3) show that the lowest yearly total cost would be obtained with the C20F membrane; which was expected given that the flux was the highest for that membrane, resulting in the smallest membrane area requirement. However, the C20F did not have the highest GGM retention and thus the cost per ton product was not the lowest. The difference of the total cost between the C20F and RC70PP was small. The RC70PP had the lowest cost per ton product because of the 30% higher GGM production compared with the C20F; in addition, the RC70PP had the second highest average flux and was thus the most cost-efficient membrane. The cost of around 48 €/ton GGM is reasonable compared with similar calculations in other studies [31,32]. Although these cost estimates are appropriate for comparing different membranes and membrane filtration setups, they are not intended for designing a plant. The estimations used assumed that the same cleaning procedure was required and thus they did not include the dynamics of fouling over time. This, however, requires long-term membrane-filtration studies and cleaning optimization on larger pilot scale, which is out of the scope for this paper. The GR51PP had the highest cost per ton product, which was because of the low average flux, given that the GGM production was in the same range compared to the hydrophilic membranes. The GR51PP also had the lowest separation degree of around 16% (highest lignin to GGM content), which made it the least favorable choice in the series. 

## 4. Conclusions

- Initial membrane filtration trials and comparison of the raw material (SSL) and retentates show that most of the solutes are in the same MW range as the cut-off specifications of the membranes. Therefore, the hydrophilic membranes with highest MWCO, experienced the highest degree of fouling.
- The fouling was due to pore blocking as has been seen previously with the hydrophobic membranes.
- The hydrophilic membranes had an overall lower lignin retention and higher GGM retention compared with the hydrophobic, possibly because of the lower lignin affinity and adsorption.
- The hydrophilic membranes were shown to be superior to the hydrophobic membranes when it comes to separation, fouling, and capacity.
- Analyzing the SSL using SEC with water as an eluent has been shown to be a promising method for the separation of lignosulfonates and LCCs from GGM due to the effect of ion exclusion in the SEC column.
- The optimal membrane (RC70PP) was compared with the GR51PP by analyzing the different retentates using SEC and 2D HSQC NMR.
- The results showed that the hydrophilic membrane retained a higher amount of polysaccharides compared with the hydrophobic membrane. This resulted in a higher portion of LCCs being retained in the hydrophilic membrane; which occurred because of the lower MWCO of the hydrophilic membrane.
- The 2D HSQC NMR indicated that the retentate from the hydrophilic membrane had a higher amount of LCCs. No other major structural differences were observed between the two retentates.
- The most cost-efficient membrane was chosen based on the flux and product yields. The C20F membrane had the lowest total costs per year; however, the RC70PP led to higher GGM production and yields and thus, the cost per ton product was the lowest for the RC70PP.

## Figures and Tables

**Table 1:** List of membranes used in the study, where MWCO denotes molecular weight cut-off.

Designation	Manufacturer	MWCO (kDa)	Membrane Material	Hydrophilicity
**GR51PP**	Alfa Laval Nordic A/S	50	Polysulfone	Hydrophobic
**C30F**	Microdyn-Nadir GmbH	30	Regenerated cellulose	Hydrophilic
**C20F**	Microdyn-Nadir GmbH	20	Regenerated cellulose	Hydrophilic
**RC70PP**	Alfa Laval Nordic A/S	10	Regenerated cellulose	Hydrophilic
**C5F**	Microdyn-Nadir GmbH	5	Regenerated cellulose	Hydrophilic

**Table 2:** Assumptions for the cost estimates (Based on Spiral module (2517) with 48 mil spacer by Alfa Laval).

Investment cost (€/m^2^)	500
Annuity factor (/year)	0.1
Membrane cost (less housing) (€/m^2^)	50
Membrane life-time (years)	1.5
Electricity price (€/MWh)	38
Cleaning Cost (€/m^2^/cycle)	0.13
Cleaning time (h/day)	1
Extra membrane area for cleaning (%)	20
Maintenance and labor costs (% of capital cost)	5
Operation Time (h/year)	8000
Pump efficiency (-)	0.8
Permeate density (kg/m^3^)	1100
Maximum pressure drop (at 1.5 m^3^/h) (bar)	0.6
Membrane area per module (m^2^)	1.2
Plant feed flow (m^3^/h)	1
Plant volume reduction (%)	90
Plant transmembrane pressure (bar)	5.5

**Table 3:** Cost estimations for galactoglucomannan (GGM) recovery using the hydrophobic and hydrophilic membranes.

Parameters	C30F	C20F	RC70PP	C5F	GR51PP
Average flux (L/m^2^ h)	105.0	147.4	132.2	82.8	83.9
GGM yield (%)	56.2	45.0	59.3	65.6	45.9
GGM produced (kg/h)	2.61	2.09	2.75	3.04	2.51
Membrane Area (m^2^)	7.79	5.55	6.19	9.89	9.75
Feed pump energy demand (kW)	0.17	0.17	0.17	0.17	0.17
Recirculation pump energy demand (kW)	0.20	0.14	0.16	0.26	0.25
Capital cost (€/year)	468	333	371	593	585
Electricity cost (€/year)	114	97	102	131	130
Membrane replacement cost (€/year)	312	222	247	395	390
Cleaning cost (€/year)	405	288	321	514	507
Maintenance and labor costs (€/year)	23	17	19	30	29
**Total cost (€/year)**	**1322**	**957**	**1060**	**1663**	**1641**
**Cost per ton product (€/ton GGM)**	**63.36**	**57.26**	**48.23**	**68.31**	**81.86**
